# Biosynthesis of Two Types of Exogenous Antigenic Polysaccharides in a Single *Escherichia coli* Chassis Cell

**DOI:** 10.3390/life15060858

**Published:** 2025-05-26

**Authors:** Jingjing Hao, Haoqian Liao, Shuhong Meng, Yan Guo, Li Zhu, Hengliang Wang, Yufei Lyu

**Affiliations:** 1 College of Food Science and Technology, Shanghai Ocean University, Shanghai 201306, China; 2 State Key Laboratory of Pathogen and Biosecurity, Beijing Institute of Biotechnology, Beijing 100071, China

**Keywords:** *Escherichia coli*, *Klebsiella pneumoniae*, biosynthesis, polysaccharides, universal chassis cells, combination of two types of glycoproteins

## Abstract

*Escherichia coli* and *Klebsiella pneumoniae* are major contributors to the global challenge of antimicrobial resistance, posing serious threats to public health. Among current preventive strategies, conjugate vaccines that utilize bacterial surface polysaccharides have emerged as a promising and effective approach to counter multidrug-resistant strains. In this study, both the Wzy/Wzx-dependent and ABC transporter-dependent biosynthetic pathways for antigenic polysaccharides were introduced into *E. coli* W3110 cells. This dual-pathway engineering enabled the simultaneous biosynthesis of two structurally distinct polysaccharides within a single host, offering a streamlined and potentially scalable strategy for vaccine development. Experimental findings confirmed that both polysaccharide types were successfully produced in the engineered strains, although co-expression levels were moderately reduced. A weak competitive interaction was noted during the initial phase of induction, which may be attributed to competition for membrane space or the shared use of activated monosaccharide precursors. Interestingly, despite a reduction in plasmid copy number and transcriptional activity of the biosynthetic gene clusters over time, the overall polysaccharide yield remained stable with prolonged induction. This suggests that extended induction does not adversely affect final product output. Additionally, two glycoproteins were efficiently generated through in vivo bioconjugation of the synthesized polysaccharides with carrier proteins, all within the same cellular environment. This one-cell production system simplifies the workflow and enhances the feasibility of generating complex glycoprotein vaccines. Whole-cell proteomic profiling followed by MFUZZ clustering and Gene Ontology analysis revealed that core biosynthetic genes were grouped into two functional clusters. These genes were predominantly localized to the cytoplasm and were enriched in pathways related to translation and protein binding. Such insights not only validate the engineered biosynthetic routes but also provide a molecular basis for optimizing future constructs. Collectively, this study presents a robust synthetic biology platform for the co-expression of multiple polysaccharides in a single bacterial host. The approach holds significant promise for the rational design and production of multivalent conjugate vaccines targeting drug-resistant pathogens.

## 1. Introduction

Pathogenic bacteria—particularly multidrug-resistant (MDR) strains—have emerged as a major threat to global health, rivaling that of viral pathogens. Although the clinical introduction of antibiotics revolutionized the treatment of infectious diseases, it has also inadvertently accelerated the adaptive evolution of pathogens, giving rise to widespread antimicrobial resistance. In recent years, there has been an alarming global increase in infections caused by MDR and extensively drug-resistant bacteria, including *Escherichia coli*, *Mycobacterium tuberculosis*, *Acinetobacter baumannii*, and *Klebsiella pneumoniae*, among others [1,2,3]. According to projections on antimicrobial resistance (AMR), without additional interventions, antimicrobial resistance could lead to 10 million deaths annually by 2050 [4].

Vaccination remains one of the most effective approaches to combat MDR bacterial infections. Among various strategies, polysaccharide conjugate vaccines have demonstrated notable success in preventing infectious diseases such as bacterial meningitis and pneumonia [5]. These vaccines target bacterial surface polysaccharides, particularly the lipopolysaccharide (LPS) layer, which comprises lipid A, core oligosaccharide chains, and a highly variable O-specific polysaccharide (O-antigen) component. Due to their surface exposure and high variability, O-antigenic polysaccharides (OPS) are key antigenic targets in vaccine development. In polysaccharide conjugate vaccine platforms, OPS are covalently linked to carrier proteins, transforming them into T cell-dependent antigens. This design elicits a robust immune response by engaging T cells, facilitating immune memory formation and antibody class switching. As a result, high-affinity, complement-activating IgG antibodies with enhanced effector functions are generated [6].

Despite their clinical promise, the development of polysaccharide conjugate vaccines is often hampered by the inherent limitations of traditional chemical conjugation techniques. These methods involve labor-intensive purification procedures and complex synthesis steps, significantly increasing production costs [7]. However, advances in the molecular understanding of prokaryotic glycosylation systems—such as the protein glycosyltransferase PglB and related enzymes—have led to the development of innovative biosynthetic platforms [8]. These systems leverage the bacterial periplasmic space for in vivo assembly of conjugate vaccines, integrating glycan biosynthesis and enzymatic protein conjugation into a single cellular framework [9,10,11]. This spatial and temporal integration streamlines the production process by eliminating the need for multistep purification and addressing challenges such as steric hindrance and reaction specificity associated with traditional methods [12].

**Figure 1:**
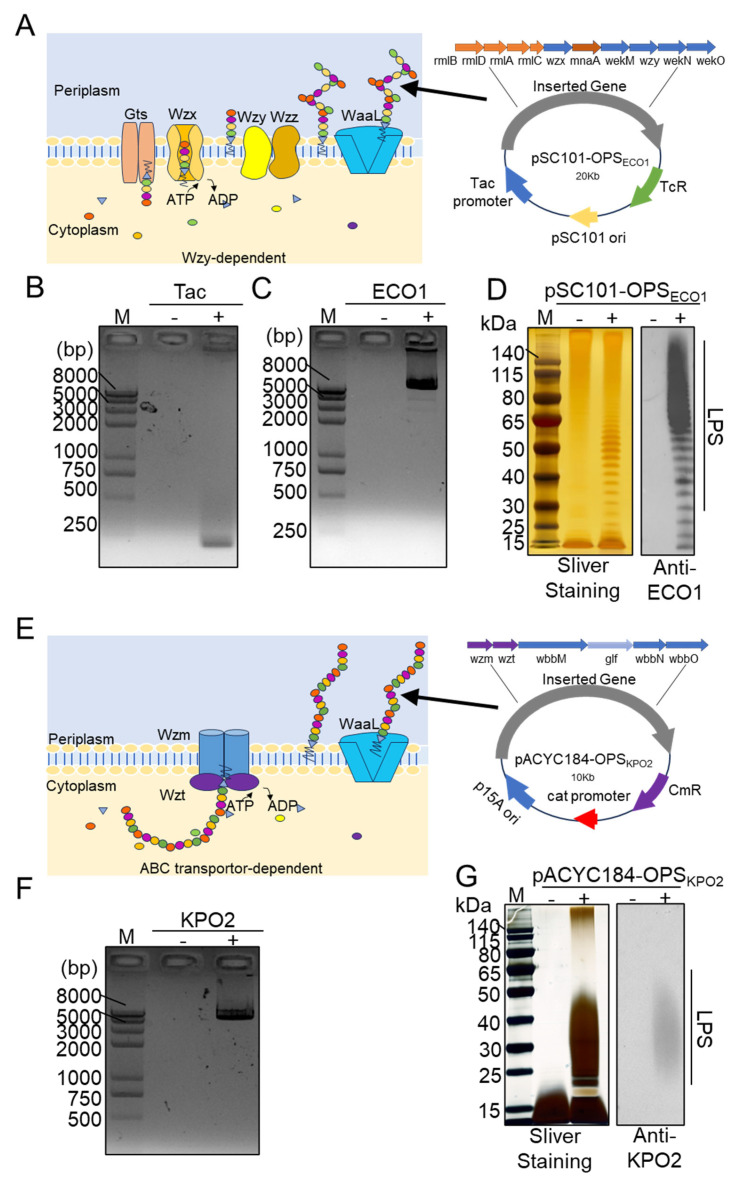
Construction and characterization of the ECO1 and KPO2α biosynthetic polysaccharide plasmid. (**A**) Depiction of the O-polysaccharide antigen assembly Wzy/Wzx pathway of ECO1 and the map of the plasmid pSC101-OPS_ECO1_. (**B**) Colony PCR to validate the tac fragment on pSC101-tac (negative control was pSC101 plasmid). (**C**) Colony PCR to verify the *ECO1* cluster fragment on pSC101-tac-OPS_ECO1_. (**D**) Characterization of ECO1 LPS by silver staining and western blotting using anti-ECO1 serum as the primary antibody. (**E**) Diagram of the O-polysaccharide antigen assembly ABC pathway of KPO2α and the map of the plasmid pACYC184-OPS_KPO2α_. (**F**) The KPO2α cluster fragment on the plasmid pACYC184-OPS_KPO2α_ was verified by colony PCR. (**G**) Analysis of pACYC184-OPS_KPO2α_ LPS by silver staining and western blotting using anti-KPO2α serum as the primary antibody.

OPS, which serve as essential molecular signatures for immune recognition, are synthesized through two primary pathways. The Wzy/Wzx-dependent pathway is the predominant route for O-antigen biosynthesis in Gram-negative bacteria. In this pathway, glycan repeat units—typically composed of 3–5 distinct monosaccharides—are assembled on the cytoplasmic face of the inner membrane by glycosyltransferases. These units are then flipped into the periplasmic space via the Wzx flippase. Once in the periplasm, polymerization of the repeat units into OPS chains is catalyzed by the polymerase Wzy. The chain length of these polysaccharides is tightly regulated by the chain length determinant protein Wzz, ensuring consistent antigenic structures [13] (Figure 1A). Alternatively, the ABC transporter-dependent pathway involves the cytoplasmic elongation of OPS chains composed of a single oligosaccharide repeat unit. These fully assembled structures are then translocated across the inner membrane into the periplasm by ABC transporters (Figure 1E) [14]. The potential to integrate these two biosynthetic pathways offers a promising route to develop orthogonal, multivalent conjugate vaccines. Such a strategy could elicit broad-spectrum immune responses against multiple bacterial pathogens simultaneously, highlighting the translational potential of this approach in next-generation vaccine development.

The *Escherichia coli* O1 (ECO1) serotype is a prominent representative of both enterohemorrhagic and enterotoxigenic *E. coli* strains and is associated with severe gastrointestinal illnesses, including acute gastroenteritis and hemolytic uremic syndrome. Certain ECO1 isolates also carry the NDM-1 carbapenemase gene, which confers resistance to carbapenems and other antibiotics, significantly complicating clinical management [15,16,17,18,19]. Similarly, *K. pneumoniae* is an opportunistic pathogen responsible for a wide range of infections, including pneumonia, meningitis, suppurative liver abscesses, and urinary tract infections. Alarmingly, its drug resistance rate has been reported to reach 35.2% and continues to rise globally [20,21,22,23,24]. Current research efforts have primarily focused on the biosynthesis of surface polysaccharides from individual pathogens, such as ECO1 or *Klebsiella pneumoniae* O2 (KPO2α). However, little attention has been given to the simultaneous expression of two distinct exogenous polysaccharides within a single engineered bacterial host, leaving a significant gap in the development of broad-spectrum vaccine platforms.

The complexity of bacterial polysaccharide biosynthesis continues to pose substantial challenges to the advancement of polysaccharide conjugate vaccines. The term “conjugate” refers to the covalent linkage of polysaccharide antigens to carrier proteins, thereby enhancing immunogenicity and enabling the induction of pathogen-specific immune responses. A combined conjugate vaccine, capable of targeting multiple bacterial strains within a single formulation, offers substantial advantages in public health settings by broadening protective coverage with fewer immunizations. However, traditional vaccine production processes remain technically demanding [25,26], typically involving strain fermentation followed by labor-intensive, stepwise purification of individual antigens. To address these challenges, the development of a single engineered strain capable of synthesizing multiple vaccine-relevant polysaccharides simultaneously offers a promising strategy. Such an approach could streamline production, shorten fermentation timelines, and reduce manufacturing complexity. Nonetheless, technical hurdles remain, including metabolic burden, genetic compatibility between biosynthetic clusters, and competition for cellular resources.

In this study, we introduced both the Wzy/Wzx-dependent and ABC transporter-dependent biosynthetic pathways for antigenic polysaccharides into *Escherichia coli* W3110 cells. Our results demonstrate that this engineered strain is capable of co-synthesizing two structurally distinct polysaccharides with high efficiency. Although minor competition between the pathways was observed during early induction—likely due to membrane space constraints or competition for shared activated sugar substrates—these effects diminished over time. Interestingly, extended induction was associated with a decline in plasmid copy number and reduced transcription of the biosynthetic cluster genes; however, polysaccharide yields remained stable. Furthermore, we successfully produced two distinct glycoproteins in vivo by enzymatically coupling the expressed polysaccharides to carrier proteins within the same cell. These findings represent a significant advancement toward a more efficient, integrated platform for the concurrent biosynthesis of multiple polysaccharide antigens, providing a streamlined strategy for polyvalent conjugate vaccine development.

## 2. Materials and Methods

### 2.1. Strains and Plasmids

The strains and plasmids used in this study are listed in Table 1, and the primers employed in the various experiments are detailed in Table S1 (see Supplementary File). *E. coli* W3110 and its derivative strains were cultured in Luria–Bertani (LB) liquid medium containing 10 g/L tryptone, 10 g/L sodium chloride, and 5 g/L yeast extract, or on solid LB medium supplemented with 1.5% agar. To ensure plasmid stability and maintain selective pressure, chloramphenicol, ampicillin, kanamycin, and tetracycline were each added at a concentration of 50 μg/mL.

### 2.2. Construction and Electrotransformation of Biosynthetic Polysaccharide Plasmid

To enable the expression of the ECO1 OPS in *E. coli*, a gene cluster approximately 10 kb in length was cloned into the pSC101-tac plasmid, resulting in the recombinant construct pSC101-OPS_ECO1_. The construction process was performed in two stages. First, the tac promoter, an inducible expression element, was inserted into the pSC101-tac plasmid. Colony polymerase chain reaction (PCR) was then used to confirm the successful integration of the promoter sequence. Next, the pSC101-tac plasmid backbone was linearized via restriction digestion, and the ECO1 gene cluster was amplified by PCR. The linearized vector and PCR-amplified gene cluster were assembled using homologous recombination, yielding the final pSC101-OPS_ECO1_ plasmid.

An analogous strategy was employed to construct the pACYC184-OPS_KPO2α_ plasmid, in which the promoter and gene cluster encoding the KPO2α OPS were inserted into the pACYC184 backbone.

For transformation, 250–300 ng of the constructed plasmid DNA was mixed with competent *E. coli* W3110 and W3110ΔΔ cells and transferred into a 2 mm electroporation cuvette. The cuvette was chilled on ice for 5 min before electroporation, which was performed at 2500 V. Immediately afterward, ice-cold SOC medium was added to the cells, which were then incubated at 37 °C for 1–2 h to allow for recovery. The entire culture was plated onto LB agar containing the appropriate antibiotics and incubated overnight at 37 °C. Resulting colonies were subjected to PCR verification to confirm successful plasmid integration.

### 2.3. Induced Biosynthesis of Exogenous LPS and Glycoprotein

To initiate the biosynthesis of LPS and glycoproteins, bacterial strains were revived from frozen stocks using inoculation loops and streaked onto LB agar plates, followed by overnight incubation at 37 °C. The next day, single colonies were selected for PCR confirmation. Once validated, a confirmed colony was inoculated into 5 mL of LB broth containing the appropriate antibiotic. The starter culture was diluted 1:100 into fresh LB medium and incubated at 37 °C at 220 rpm for 2–3 h until the optical density at 600 nm (OD_600_) reached 0.6–0.8. At this point, isopropyl-β-D-thiogalactopyranoside (IPTG) was added to the culture to a final concentration of 1 mmol/L to induce gene expression. The cultures were then transferred to a shaker and incubated at 30 °C at 220 rpm for 10–12 h to facilitate the biosynthesis of the target LPS and glycoproteins.

### 2.4. LPS Extraction

After cultivation, the bacterial cultures were centrifuged, and the resulting cell pellets were washed three times with pre-chilled distilled water. The pellets were weighed and resuspended in distilled water at a ratio of 1 g of pellet per 3 mL of water. The resuspended cells underwent three cycles of alternating ice and hot water baths. Following this treatment, an equal volume of 90% phenol was added to the cell suspension, and the mixture was incubated in a water bath at 68 °C for 30 min. The aqueous phase was collected, and the extraction process was repeated to ensure efficient recovery of LPSs. The combined aqueous extracts were dialyzed extensively against distilled water to remove residual phenol. The dialysate was then treated with DNase I and RNase A at final concentrations of 5 μg/mL each and incubated at 37 °C for 3 h to remove nucleic acids. Subsequently, protein contamination was eliminated by adding proteinase K (Vazyme, Nanjing, China) at a final concentration of 20 μg/mL, followed by incubation at 60 °C for 1 h. The mixture was then boiled for 10 min and centrifuged at 8000 rpm for 20 min. The resulting supernatant, which contained purified LPS, was collected for downstream analyses.

### 2.5. Silver Staining

Purified LPS samples were mixed with an equal volume of 2× sodium dodecyl sulfate (SDS) loading buffer and heated in boiling water for 10 min. The samples were then separated by sodium dodecyl sulfate–polyacrylamide gel electrophoresis (SDS-PAGE) (Genscript, Zhenjiang, China). The SDS loading buffer consisted of 100 mM Tris-HCl (pH 6.8), 3.2% (*w/v*) SDS, 0.04% (*w/v*) bromophenol blue, 16% (*v/v*) glycerol, and 40 mM D,L-dithiothreitol. Following electrophoresis, the gel was rinsed in distilled water for 5 min with gentle shaking, and this wash step was repeated once. The gel was then washed with a solution of 30% ethanol and 10% acetic acid for 15 min, followed by a second identical wash. Next, the gel was rinsed with 10% ethanol for 5 min and then with distilled water for another 5 min. Silver staining was performed using reagents prepared from the Pierce^TM^ Silver Stain Kit (Thermo Fisher Scientific, Waltham, MA, USA). The gel was incubated with the sensitizing solution for 1 min, followed by a 1 min rinse with distilled water. It was then treated with the enhancement solution for 30 min and rinsed with distilled water for 20 s. Color development was initiated by adding the chromogenic solution; bands gradually appeared within 2–3 min. Color development was terminated immediately by rinsing the gel with distilled water. The stained gel was imaged, and the results were documented using an imaging system.

### 2.6. Protein Purification

Cultures induced with IPTG were harvested by centrifugation, and the cell pellets were resuspended in Buffer A (0.5 M NaCl, 10 mM imidazole, 20 mM Tris-HCl, pH 7.5). The cells were disrupted using a high-pressure homogenizer, and the lysates were centrifuged at 8000 rpm for 30 min at 4 °C. The supernatant, containing the soluble protein fraction, was collected, while the pellet was discarded. The clarified lysate was loaded onto a pre-equilibrated nickel affinity column (HisTrap HP, Cytiva, Uppsala, Sweden) using Buffer A at a flow rate of 4 mL/min. After washing the column with ten column volumes of Buffer A to remove unbound proteins, the target protein was eluted using Buffer B (0.5 M NaCl, 0.5 M imidazole, 20 mM Tris-HCl, pH 7.5). The eluate was dialyzed overnight against Q:A buffer (20 mM Tris-HCl, pH 7.0) to prepare for further purification. Anion exchange chromatography was performed using a HiTrap Q HP column (Cytiva, Sweden). The column was equilibrated with Q:A buffer, and proteins were eluted using a linear gradient of Q:A to Q:B buffer (20 mM Tris-HCl, 1 M NaCl, pH 7.0). Fractions containing the glycoprotein were collected and analyzed by SDS-PAGE followed by Coomassie Brilliant Blue staining for identification.

### 2.7. Western Blotting (WB)

Specific polysaccharides were detected by incubating the transfected membranes with KPO2α- and ECO1-specific sera (diluted 1:1000) as primary antibodies, as described previously [27]. A goat anti-rabbit secondary antibody conjugated to horseradish peroxidase, diluted 1:5000 (Transgen Biotech, Beijing, China), was then applied and incubated at room temperature for 30 min. This was followed by three 10 min washes with TBST. Membranes were subsequently imaged using the ChemiDoc MP Imaging System (Bio-Rad, Hercules, CA, USA) in the presence of a chemiluminescent substrate. To evaluate glycoprotein expression, a horseradish peroxidase-conjugated anti-His antibody (diluted 1:1500, Abmart, Shanghai, China) was used for detection and imaging.

### 2.8. Quantitative PCR Analysis

Quantitative polymerase chain reaction (qPCR) was employed to monitor variations in copy number and transcription levels of glycoprotein gene clusters over time, relative to reference genes, in cells harboring two different plasmids. Genomic DNA and total RNA were isolated from W3110/pSC101-OPS_ECO1_+pACYC184-OPS_KPO2α_ cells at various time points following IPTG induction. Genomic DNA contamination in total RNA samples was removed using the HiFiScript gDNA Removal Kit. Before reverse transcription, total RNA was tested by PCR amplification using primers specific to the 16S rRNA and 23S rRNA genes to confirm the absence of DNA contamination. Double-stranded cDNA was synthesized using a commercial cDNA synthesis kit. qPCR and reverse transcription-quantitative polymerase chain reaction (RT-qPCR) were performed using 2× SYBR Mix (Yeasen, Shanghai, China) and the CFX96 Connect Real-Time PCR System (Bio-Rad, Hercules, CA, USA). The specific primers used for RT-qPCR are listed in Table S1, and the final primer concentration was 0.5 µM. Relative gene expression and plasmid copy number changes were calculated using the delta Ct method, with *gapA*, a chromosomal gene, serving as the internal control.

### 2.9. Mass Spectrometry Analysis

To investigate the differential glycoprotein expression driven by distinct glycoprotein biosynthesis gene clusters using a common carrier protein within the same cellular environment, we conducted label-free liquid chromatography–tandem mass spectrometry on whole-cell extracts collected at various time points following IPTG induction, as previously described (Reference LFQ Quantitative Proteomic Analysis Report).

Protein concentrations were determined using the 2D Quant Kit. Following extraction, proteins were concentrated using an ultrafiltration tube (0.5 mL 10 kDa, Thermo Fisher Scientific) and washed three times with 8 M urea. Alkylation was performed with 20 mM iodoacetamide for 30 min at room temperature in the dark. Residual iodoacetamide was removed by washing with 8 M urea, followed by three washes with 50 mM ammonium bicarbonate to eliminate the remaining urea. Proteins were digested in-column with trypsin at 37 °C for 13 h. After centrifugation, the resulting peptides were transferred to clean tubes, vacuum-dried in the presence of 100% formic acid, and reconstituted in 0.1% formic acid. The peptide mixtures were analyzed using a nano-liquid chromatography system coupled with an Orbitrap Lumos Fusion mass spectrometer (Thermo Fisher Scientific). Acquired spectra were searched against the *E. coli* W3110 genome from GenBank, along with three plasmid reference sequences (pSC101-OPS_ECO1_, pACYC184-OPS_KPO2α_, and pET28a-sc), using the Proteome Discoverer 2.5 software (Thermo Fisher Scientific).

Functional annotation and enrichment analysis of significant gene clusters associated with biological processes (BPs), molecular functions (MFs), and cellular components (CCs) were performed using Gene Ontology (GO) enrichment analysis on the DAVID database through the Omicsolution platform (https://davidbioinformatics.nih.gov/home.jsp, accessed on 24 May 2025) [28].

## 3. Results

### 3.1. Construction of Two Polysaccharides in the E. coli System

As illustrated in Figure 1A, the synthesis of the O-antigen polysaccharide in ECO1 proceeds via the Wzy/Wzx-dependent pathway. To enable the production of the ECO1 polysaccharide, the low-copy plasmid pSC101 was sequentially engineered to generate pSC101-OPS_ECO1_, which incorporates the inducible tac promoter and the complete ECO1 O-antigen gene cluster. Each modification step was validated by colony PCR using primers specific to the tac promoter (Figure 1B) and the ECO1 gene cluster (Figure 1C).

The host strain *E. coli* W3110, which carries an inactive *wbbL* gene encoding a rhamnose-transferase, is unable to synthesize its own OPS and thus serves as a suitable chassis for heterologous OPS biosynthesis. The engineered plasmid pSC101-OPS_ECO1_ was transformed into *E. coli* W3110, and the original W3110 strain was used as a negative control to verify exogenous polysaccharide production. Following the induction of gene expression in both W3110/pSC101-OPS_ECO1_ and W3110, LPSs were extracted from each strain. The LPS samples were separated by SDS-PAGE and analyzed via silver staining and WB using ECO1-specific antiserum as the primary antibody (Figure 1D). Silver staining revealed a distinct ladder-like banding pattern in the LPS of W3110/pSC101-OPS_ECO1_, in contrast to the original W3110 strain, indicating successful synthesis of the exogenous polysaccharide. Furthermore, WB confirmed the specific binding of ECO1 serum antibodies to the LPS from the recombinant strain, while no binding was observed with LPS from the unmodified W3110 strain.

The synthesis of the OPS for KPO2α is mediated via the ABC transporter-dependent pathway (Figure 1E). Similar to the strategy employed for ECO1, we cloned the KPO2α gene cluster into the plasmid vector pACYC184, generating the recombinant plasmid pACYC184-OPS_KPO2α_. PCR analysis using primers specific to the KPO2α gene cluster, with the unmodified pACYC184 vector serving as a negative control, confirmed the successful incorporation of the desired gene fragments into pACYC184-OPS_KPO2α_ (Figure 1F). This plasmid was subsequently introduced into *E. coli* W3110. Both recombinant (W3110/pACYC184-OPS_KPO2α_) and parental (W3110) strains were cultured under conditions promoting the expression of the KPO2α gene cluster. Following LPS extraction, the samples were separated by SDS-PAGE and subjected to silver staining and WB using KPO2α-specific antiserum. The recombinant strain exhibited distinct ladder-like bands compared to the W3110 control, as revealed by both silver staining and WB (Figure 1G), confirming the successful biosynthesis of KPO2α OPS in *E. coli* W3110.

### 3.2. Construction of Co-Expression of Two Polysaccharides in E. coli System

**Figure 2:**
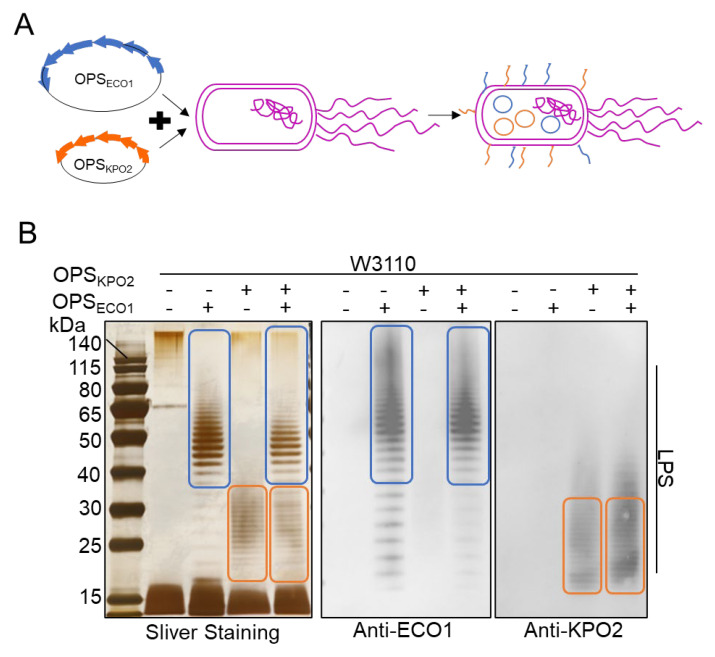
Characterization of two types of exogenous polysaccharide biosynthesis systems. (**A**) Schematic diagram of two types of polysaccharides biosynthesis systems. (**B**) LPS analysis of W3110/pSC101-OPS_ECO1_+pACYC184-OPS_KPO2α_ by silver staining and western blotting using KPO2α and ECO1 serum as the primary antibody, respectively. The blue box is the band of ECO1 LPS. The orange box represents the band of KPO2α LPS.

Given that the exogenous polysaccharides ECO1 and KPO2α can be independently synthesized in *E. coli*, we further investigated whether LPS synthesis in *E. coli* could be achieved through the simultaneous operation of two O-antigen polysaccharide biosynthesis pathways—the Wzy/Wzx-dependent pathway and the ABC transporter-dependent pathway. To this end, the plasmid pACYC184-OPS_KPO2α_ was introduced into the W3110/pSC101-OPS_ECO1_ strain via electroporation, and the resulting strain was designated W3110/pSC101-OPS_ECO1_+pACYC184-OPS_KPO2α_ (Figure 2A).

To evaluate the capability of these strains to simultaneously express both polysaccharides, we performed a comparative analysis of LPS expression among *E. coli* strains W3110, W3110/pACYC184-OPS_KPO2α_, W3110/pSC101-OPS_ECO1_, and W3110/pSC101-OPS_ECO1_+pACYC184-OPS_KPO2α_. LPS was extracted 10 h after IPTG induction of OPS_ECO1_ expression and analyzed via SDS-PAGE followed by silver staining and WB. Antisera against both ECO1 and KPO2α were used as primary antibodies. As shown in Figure 2B, silver staining of the recombinant strain W3110/pSC101-OPS_ECO1_+pACYC184-OPS_KPO2α_ revealed ladder-like bands corresponding to the OPSs of both ECO1 (indicated by the blue circle) and KPO2α (indicated by the orange circle). WB further demonstrated that LPS from this strain reacted specifically with both anti-ECO1 and anti-KPO2α antibodies, confirming successful co-expression of the two distinct O-antigens within a single *E. coli* host. The molecular weights of the LPS bands for ECO1 and KPO2α were primarily distributed within the ranges of 50–80 kDa and 15–40 kDa, respectively. Interestingly, silver staining indicated that the expression levels of both ECO1 and KPO2α LPS were reduced in the dual-expression strain compared to the levels observed when each polysaccharide was expressed individually (Table S2, Supplementary File). This observation raises questions regarding potential factors that may limit the efficient and simultaneous biosynthesis of two distinct O-antigen polysaccharides in the W3110/pSC101-OPS_ECO1_+pACYC184-OPS_KPO2α_ system.

### 3.3. Effect of IPTG Induction Time on Simultaneous Biosynthesis of Two Types of Polysaccharides

**Figure 3:**
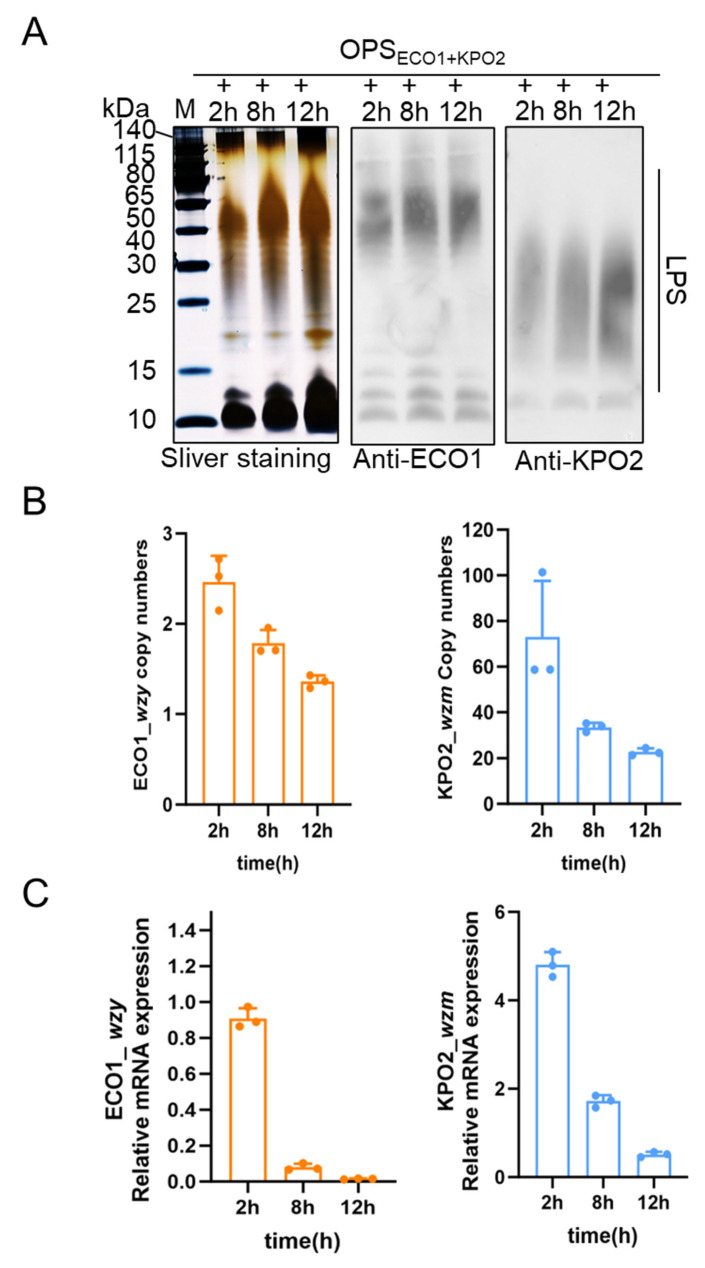
Analysis of biosynthetic LPS of W3110/pSC101-OPS_ECO1_+pACYC184-OPS_KPO2α_ induced by IPTG at different times. (**A**) Expression analysis of KPO2α LPS at different culture times and ECO1 LPS at different induction times by silver staining and WB. (**B**) Gene copy numbers of pSC101-OPS_ECO1_ and pACYC184-OPS_KPO2α_ under different induction and culture times. (**C**) mRNA transcription levels of pSC101-OPS_ECO1_ and pACYC184-OPS_KPO2α_ under different induction and culture times.

To investigate the factors influencing the simultaneous synthesis of two types of polysaccharides, LPS samples from the strain W3110/pSC101-OPS_ECO1_+pACYC184-OPS_KPO2α_ were collected at different time points (2, 8, and 12 h) following IPTG induction and subjected to silver staining and WB analysis. As shown in Figure 3A, the silver staining results corroborated earlier findings. Western blot analysis further confirmed the presence of both ECO1- and KPO2α-specific LPS, as both antigens were detectable at all examined time points. Notably, LPS expression was evident as early as 2 h post-induction, indicating robust early-stage activity of both O-antigen biosynthesis pathways within a single *E. coli* cell. Densitometric analysis using ImageJ 1.54g software revealed that the expression level of ECO1-derived LPS at 2 h and 8 h post-induction was significantly higher than that of KPO2α-derived LPS (Table S2, Supplementary File). However, the expression of KPO2α LPS gradually increased with prolonged induction, and by 12 h, there was no significant difference in the expression levels of the two LPS types. These results suggest that although both polysaccharide biosynthesis pathways are active, their expression kinetics differ, with ECO1 being expressed more rapidly than KPO2α. Importantly, no evidence of synergistic enhancement or competitive inhibition between the two O-antigen biosynthesis pathways was observed.

To further investigate potential underlying genomic and transcriptional factors, genomic DNA and total RNA were extracted from W3110/pSC101-OPS_ECO1_+pACYC184-OPS_KPO2α_ at the same time points. The relative plasmid copy numbers of pSC101-OPS_ECO1_ and pACYC184-OPS_KPO2α_ were quantified using *gapA*, a chromosomal housekeeping gene in *E. coli* W3110, as an internal control; *wzy* in pSC101-OPS_ECO1_ and *wzm* in pACYC184-OPS_KPO2α_ were selected as representative markers for each plasmid, with primer sequences listed in Table S1. qPCR analysis (Figure 3B) showed a time-dependent decrease in the copy numbers of both plasmids. Notably, the copy number of pACYC184-OPS_KPO2α_ consistently remained higher than that of pSC101-OPS_ECO1_ throughout the induction period.

This trend suggests a potential gradual loss or instability of the plasmids during extended culture, particularly under the stress of prolonged induction. To evaluate gene expression at the transcriptional level, we quantified the mRNA levels of *wzy* and *wzm* at the same time points using RT-qPCR, again using *gapA* as the reference gene. The results (Figure 3C) demonstrated a similar declining trend in mRNA levels over time for both plasmids. Consistent with the genomic analysis, the transcription level of *wzm* (from pACYC184-OPS_KPO2α_) was significantly higher than that of *wzy* (from pSC101-OPS_ECO1_) across all time points. Together, these data suggest that both plasmid stability and transcriptional activity contribute to the differential expression of the two LPS types. The observed reduction in plasmid copy number and mRNA levels over time may partially account for the changes in LPS expression dynamics and the convergence of expression levels by 12 h post-induction. (The data in Figure 3B,C are in Table S3.)

### 3.4. Construction and Characterization of SC-OPS_ECO1+KPO2α_

**Figure 4:**
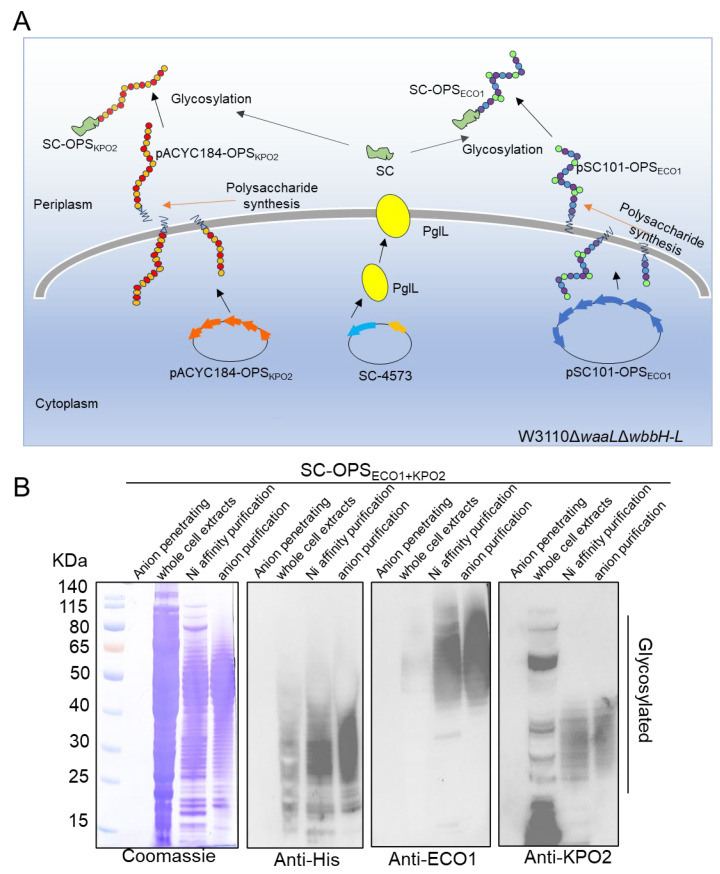
Diagram design and biosynthesis of glycoprotein. (**A**) Schematic diagram of SC-OPS_ECO1+KPO2α_ glycoprotein synthesis. (**B**) Anionic column penetrating samples, whole cell extracts, glycoproteins purification by nickel column, and glycoproteins purification by anionic column were characterized by Coomassie blue staining and western blotting using anti-His, anti-ECO1, and anti-KPO2α antibodies.

Building upon the successful co-expression of two polysaccharides within a single cellular system, we next aimed to produce the SC-OPS_ECO1+KPO2α_ glycoprotein. This was accomplished by sequentially introducing three plasmids into competent *E. coli* W3110ΔwaaLΔwbbH-L cells: pSC101-OPS_ECO1_, which drives the biosynthesis of the ECO1polysaccharide; pACYC184-OPS_KPO2α_, which facilitates the synthesis of the KPO2α polysaccharide; and pET-28a-*pglL*-SC-4573, which encodes the glycosyltransferase PglL, the carrier protein SpyCatcher, the glycosylation sequence 4573 [29], and a 6×His tag (Figure 4A). The engineered W3110ΔΔ strain lacks *waaL*, which abolishes the attachment of OPS to the lipid A-core, thereby preventing native LPS assembly. In addition, deletion of *wbbH-L* reduces endogenous polysaccharide synthesis, minimizing competition and enhancing the incorporation of exogenous polysaccharides.

As described in the Materials and Methods section, the validated recombinant strain was cultured, and whole-cell extracts were obtained. Target glycoproteins were subsequently purified using nickel affinity chromatography followed by anion exchange chromatography, resulting in enhanced purity of the final product.

Whole-cell extracts, glycoproteins isolated via nickel affinity chromatography, and glycoproteins further purified by anion exchange chromatography—along with the penetrating protein used as a negative control—were analyzed using Coomassie blue staining and WB with anti-His, anti-ECO1, and anti-KPO2α antibodies.

Coomassie blue staining (Figure 4B) revealed that the purity of the target glycoprotein increased progressively across the purification steps. When the two polysaccharide gene clusters were co-expressed in the same cells along with PglL and SpyCatcher, the resulting glycoprotein displayed a characteristic ladder band pattern in WB analysis using the His antibody. This pattern indicates the successful conjugation of the two types of OPS to SpyCatcher, forming the SC-OPS_ECO1+KPO2α_ glycoprotein via the catalytic activity of glycosyltransferase PglL. Although the ECO1 glycoprotein, which has an estimated molecular weight of 50–80 kDa, was not visible, this may be due to the large size of the glycoprotein hindering the efficient binding of the anti-His antibody. The ladder bands, corresponding to both glycoproteins and falling within the molecular weight range of LPS, were further evaluated using anti-ECO1 and anti-KPO2α antibodies. Compared with the results obtained using the anti-His antibody, both anti-ECO1 and anti-KPO2α antibodies showed relatively weak hybridization with the whole-cell extracts, particularly the anti-KPO2α antibody, which also displayed several non-specific bands.

### 3.5. Timing Analysis of SC-OPSECO1+KPO2α Biosynthesis in a Single Cell

**Figure 5:**
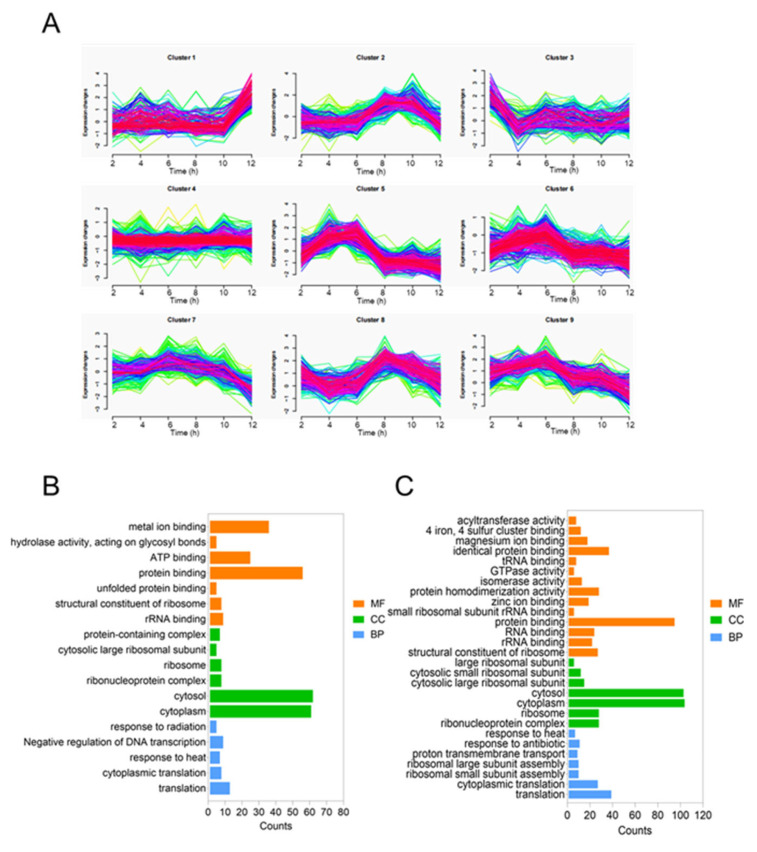
Timing analysis of SC-OPS_ECO1+KPO2α_ biosynthesis in a single cell. (**A**) MFUZZ analysis of glycoprotein synthesis proteomics. (**B**) GO analysis of glycoprotein synthesis by clusters 2. (**C**) GO analysis of glycoprotein synthesis by clusters 8.

Furthermore, two distinct types of glycoproteins were successfully conjugated to the same carrier protein within a single cell. To further investigate the proteomic changes, we performed liquid chromatography–tandem mass spectrometry analysis on whole-cell extracts collected at various time points. The resulting data were analyzed using MFUZZ clustering and GO enrichment analysis [30]. The core genes involved in polysaccharide biosynthesis were grouped into two distinct clusters. Specifically, *wzt* and *wbbO* from the KPO2α polysaccharide gene cluster, along with *rmlC* from the ECO1 polysaccharide gene cluster, were assigned to cluster 2. Meanwhile, *wbbM* and *wbbN* from the KPO2α cluster, and *wekM*, *wekO*, and *wekN* from the ECO1 cluster, were categorized into cluster 8. Both clusters 2 and 8 (Figure 5A) displayed a trend of increasing expression levels followed by a decline over time. Genes within these clusters were predominantly localized in the cytoplasm and were mainly associated with translation and protein-binding functions (Figure 5B,C). Detailed results of the MFUZZ and GO analyses are provided in Table S4 (Supplementary File).

## 4. Discussion

In the present study, we co-transformed two distinct polysaccharide biosynthesis plasmids into the genetically modified *E. coli* W3110ΔΔ strain. The first plasmid, ECO1, harbors the O-antigen polysaccharide biosynthetic gene cluster utilizing the Wzy/Wzx-dependent pathway, while the second plasmid, KPO2α, encodes the O-antigen biosynthesis machinery following the ABC transporter-dependent system. This strategy enabled the successful biosynthesis of two structurally and immunologically distinct exogenous O-antigen polysaccharides within a single bacterial host.

Subsequently, we combined the ECO1 and KPO2α plasmids with the glycosyltransferase *pglL* gene and the pET28a-sc expression plasmid encoding the carrier protein SpyCatcher4573. The resulting engineered *E. coli* W3110ΔΔ strain facilitated the in vivo assembly of glycoproteins via protein glycan coupling technology. The bivalent glycoprotein product, SC-OPS_ECO1+KPO2α_, was successfully purified and represents a key intermediate for the future development of a bivalent polysaccharide conjugate vaccine.

Several key factors were carefully considered to ensure the successful co-expression of the two polysaccharide biosynthesis pathways. Using glycan expression technology [31], we selected plasmid backbones with compatible origins of replication and distinct antibiotic resistance markers to promote plasmid stability and compatibility. Specifically, the ECO1 plasmid incorporated the pSC101 minimal replication origin and a tetracycline resistance gene, while the KPO2α plasmid used the p15A origin and a chloramphenicol resistance gene [32]. The distinct replication origins and selection markers enabled the stable coexistence of both plasmids in the host strain without competitive interference. To construct the plasmids, we employed advanced synthetic biology tools, including homologous recombination-based sequence assembly techniques [33], which accelerated the cloning process and improved the accuracy of polysaccharide gene cluster integration. Polysaccharide-specificity assays confirmed that the two antigens were independently produced in single cells, with no detectable cross-reactivity, demonstrating their high antigenic specificity. Moreover, each polysaccharide was effectively conjugated to the SpyCatcher4573 carrier protein [34]. Since the ECO1 and KPO2α antigens are synthesized via independent assembly pathways, this minimized potential interference between the two systems and avoided yield instability commonly associated with biosynthetic competition. Compared with multivalent vaccines such as the nine-valent HPV vaccine, the measles–mumps–rubella vaccine, the DTPa-HBV-IPV/Hib combination, and the DTP-HB-Hib pentavalent vaccine [35,36,37,38], which require multiple fermentation and purification cycles, our approach demonstrates the feasibility of producing multiple glycoproteins within a single engineered host. This integrated system combines glycan biosynthesis, in vivo glycosyltransferase-mediated conjugation, and carrier protein expression, offering a more efficient and cost-effective strategy for multivalent vaccine development. Our findings suggest that this streamlined protein glycan coupling technology-based platform holds significant promise for the scalable production of bivalent or multivalent polysaccharide conjugate vaccines.

Following the successful co-expression of the ECO1 and KPO2α antigens in *E. coli*, we investigated differences in their production under various induction conditions by quantifying plasmid copy number and transcriptional activity of the biosynthetic gene clusters. To achieve greater control over antigen expression, future studies will focus on establishing finely tuned regulatory systems for conditional induction. At the proteomic level, we conducted MFUZZ clustering and GO enrichment analyses to characterize the temporal expression dynamics of the two polysaccharide pathways in response to different induction durations [39,40]. MFUZZ clustering allowed us to group genes or proteins with similar expression patterns into functional clusters, thereby improving our understanding of their co-regulation and biological significance. GO enrichment further elucidated the shared features of clustered genes in terms of cellular components, molecular functions, and biological processes. These insights contribute to a deeper understanding of the proteomic correlations underlying polysaccharide biosynthesis and can guide future efforts to optimize antigen production and enhance the immunogenic potential of polysaccharide conjugate vaccines [41].

Our engineered bis-glycoprotein expression system exhibits broad applicability and high adaptability. To construct this system, only two additional polysaccharide biosynthesis plasmids with compatible replication origins are required, ensuring their stable co-expression within a single host cell. The successful production of bis-glycoproteins is confirmed through silver staining and WB using specific antibodies. To evaluate expression levels and overall yield, we initially verified the system’s function by quantifying plasmid copy numbers and mRNA transcription levels of key biosynthetic genes. This was followed by a systematic assessment of quantitative methodologies for measuring glycoprotein output. Furthermore, we plan to examine potential cross-reactivity between two identical O-antigen biosynthesis pathways to better understand their interaction mechanisms and assess any effects on biosynthetic efficiency.

Following the successful preparation of the bivalent glycoprotein, we will apply the SpyCatcher/SpyTag covalent conjugation system to assemble a bivalent glycoconjugate vaccine. In the subsequent immunological evaluation phase, we will systematically explore optimal immunization dosages, screen adjuvant candidates, and test various immunization regimens to enhance both the immunogenicity and protective efficacy of the nano-based vaccine. If proven effective, this approach could facilitate the simultaneous, fermentation-based production of glycoprotein conjugates targeting ECO1 and KPO2α within a single engineered bacterial strain. This would enable dual protection against both pathogens using a single fermentation and purification process, markedly reducing production complexity and cost. Such improvements would enhance vaccine affordability, accessibility, and scalability, offering significant advantages for public health implementation. For industrial-scale translation, cost-efficient culture media and optimized fermentation conditions must be evaluated to maximize glycoprotein yields. In addition, advanced gene-editing tools could be employed to further refine the dual-glycan expression system, reducing the metabolic burden on host cells, accelerating bacterial growth, and improving biosynthetic efficiency.

## 5. Conclusions

In this study, we successfully introduced Wzy/Wzx-dependent and ABC transporter-dependent O-antigen biosynthesis pathways into *E. coli* W3110, enabling concurrent production of two structurally distinct exogenous polysaccharides. Moreover, we demonstrated the efficient biosynthesis of two glycoproteins through the enzymatic coupling of these polysaccharides to carrier proteins within a single engineered cell. This integrated strategy offers a streamlined and cost-effective platform for the biosynthesis of dual polysaccharide antigens and lays a robust foundation for the development of bivalent nanopolysaccharide conjugate vaccines targeting ECO1and KPO2α.

## Figures and Tables

**Table 1:** Strains and plasmids used in this study.

Strains and Plasmids	Characteristic	Source
W3110	Wild-type strain of *Escherichia Coli* W3110	Laboratory stock
pACYC184-OPS_KPO2α_	Encoded O2 serotype self-promoter and OPS of *K. pneumoniae*, Cm^r^	Laboratory stock
pSC101tac	pSC101 plasmid skeleton containing tac promoter, Tc^r^	This work
pSC101-tac-OPS_ECO1_	Encoded O1 serotype OPS of *Escherichia Coli*, Tc^r^	This work
W3110Δ*waaL*Δ*wbbH-L* (W3110ΔΔ)	*waaL*, *wbbH*, *wbbI*, *wbbJ*, *wbbK*, *wbbL* genes were knocked out in the W3110 strain	Laboratory stock
pET28a-*pgIL*-*spycatcher*4573	Cloning *spycatcher*4573 gene in pET28a, Kan^r^	Laboratory stock

## Data Availability

The data presented in this study are available upon request from the corresponding author.

## Supplementary Materials

The following supporting information can be downloaded at: https://www.mdpi.com/article/10.3390/life15060858/s1. Figure S1: Original figure; Table S1: All primers used in this study; Table S2: Gray-scale analysis of PAGE using ImageJ software; Table S3: Copy numbers and mRNA; Table S4: Related data on MFUZZ and GO analysis.

## Abbreviations

The following abbreviations are used in this manuscript:

ECO1	*Escherichia coli* O1 serotype
KPO2	*Klebsiella pneumoniae* O2 serotype
W3110ΔΔ	*Escherichia coli* W3110Δ*waaL*Δ*wbbH-L*
IPTG	isopropyl-β-D-thiogalactoside
GO	Gene Ontology
LC-MS/MS	liquid chromatography–tandem mass spectrometry
IAA	iodoacetamide
MDR	multidrug-resistant
XDR	extremely drug-resistant
LPS	lipopolysaccharide
OPS	O-antigenic polysaccharides
ORI	origin of replication
