# Supplementary material for: Biosynthesis of Two Types of Exogenous Antigenic Polysaccharides in a Single Escherichia coli Chassis Cell
Source: Life (Basel). 2025 May 26;15(6):858. doi: 10.3390/life15060858 (PMC12193795; doi:10.3390/life15060858)
Supplement: Supplementary file 1 [file life-15-00858-s001.zip › Table S1.pdf]

Table. S1 All primers used in this study

**Primers**	Primer sequence（5'-3'）
pSC101-F	TTGGAGCTTGTTCATTCGCGATCTACTGCATAATTCGTGTC
pSC101-R	GATAAGCTGTCAAACATGAGCGTGTGCTTCTCAAATGC
pSC101tac-YF	CCACCGCTGAGCAATAACTA
pSC101tac -YR	AAGCAGCCCAGTAGTAGGTTG
pBR322-F	GCTGTAGGCATAGGCTTGGTCGTGTGCTTCTCAAATGC
pBR322-R	GAGGCAGACAAGGTATAGGGATCTACTGCATAATTCGTGT C
pBR322-PF	CCCTATACCTTGTCTGCCTCC
pBR322-PR	ACCAAGCCTATGCCTACAGC
pBR322tac -YF	GCTTCCTTTCGGGCTTTGTT
pBR322tac -YR	AGCAGCAGTCGCTTCACGTT
pSC101-O1-F2	ATTTCACACAGGAAACAGAATTCATGGTGAAGATACTTGT TACTGGG
pSC101-O1-R2	GCTTTGTTAGCAGCCGGATCTTACGTATAGTAAGGAACCA GTTTC
pSC101-O1-PF2	TAAGATCCGGCTGCTAACAAAGCC
pSC101-O1-PR2	GAATTCTGTTTCCTGTGTGAAATTGTTATC
pSC101-O1-YF1	TCTTGAAATGCCAGTAGAGCG
pSC101-O1-YR1	CACCACAGTAATCAGTGGAGTGA
pSC101-O1-YF4	ATGATTGTGCTTTGGTACTTGG
pSC101-O1-YR4	GCTTGCGTCAGTGGTGTAAA
pSC101-O1-F	TCGTCAACATTCTTCAGCACC
pSC101-O1-R	ACCAAGCCTATGCCTACAGC
pBR322-O1-PF	GAAACTGGTTCCTTACTATACGTAAGATCCGGCTGCTAACA AAGC
pBR322-O1-PR	CCAGTAACAAGTATCTTCACCATGAATTCTGTTTCCTGTGT GAAATTG
pBR322-O1-YF	TCCAGACTTTACGAAACACGG
pBR322-O1-YR	GCCTTACCTTCCAGAGCATT
pAC-O2 F	GCTGACCTGATAAACTTTGCCCG
pAC-O2 R	CGTTTCCTCCCCTTAATACCCATTTC
KPO2-F	GCTGCAATTATCAAACCTCTGCATG
KPO2-R	GTCCCTTCTCAGCAAGTTGGC
W3110 gapa-F	AACACATCACCGCTGGTGCGAA
W3110 gapa-R	AGTTGGTGGTGCAGGAAGCGTT
ECO1 wzy-F	TGACTCAATTGCGCATAGCA
ECO1 wzy-R	GCAAGGCGCTGTAATGAAACA
KPO2 wzm-F	CGCTTTGTCAGTCTGGGGAT
KPO2 wzm-R	AGACAAACCGACAACCGTCA
