# Supplementary figures and images for: Biosynthesis of Two Types of Exogenous Antigenic Polysaccharides in a Single Escherichia coli Chassis Cell
Source: Life (Basel). 2025 May 26;15(6):858. doi: 10.3390/life15060858 (PMC12193795; doi:10.3390/life15060858)

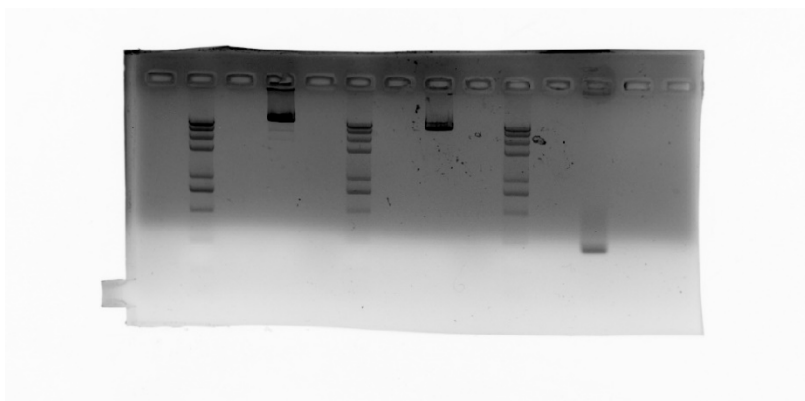

F1. C、F、B

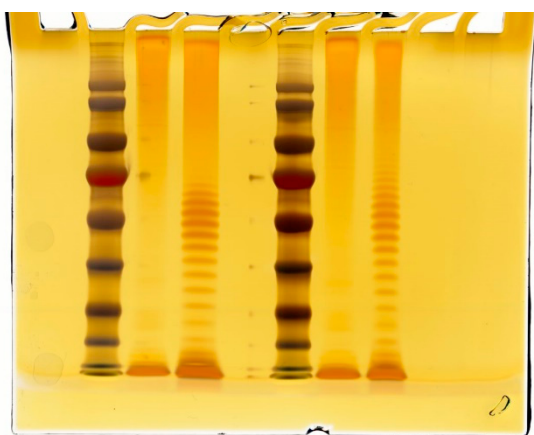

F1. D

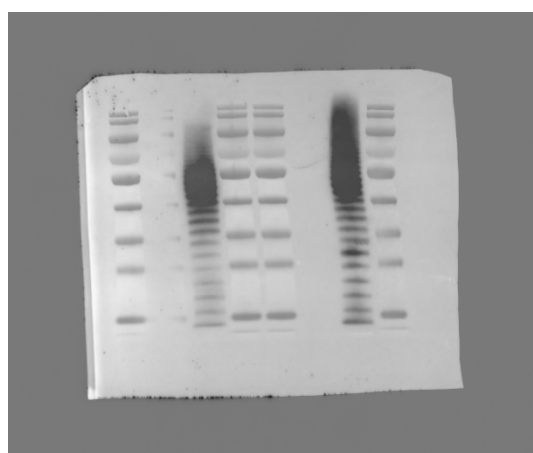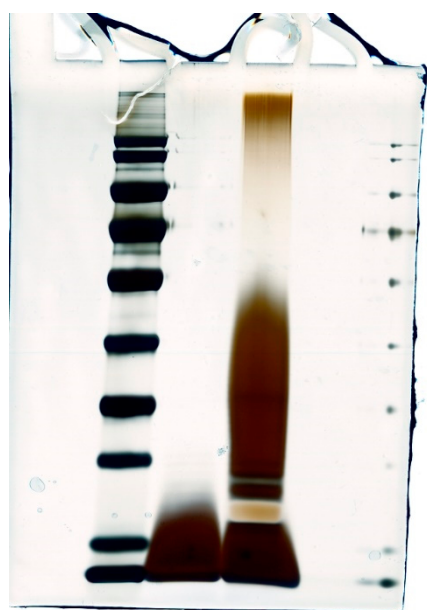

F1. G

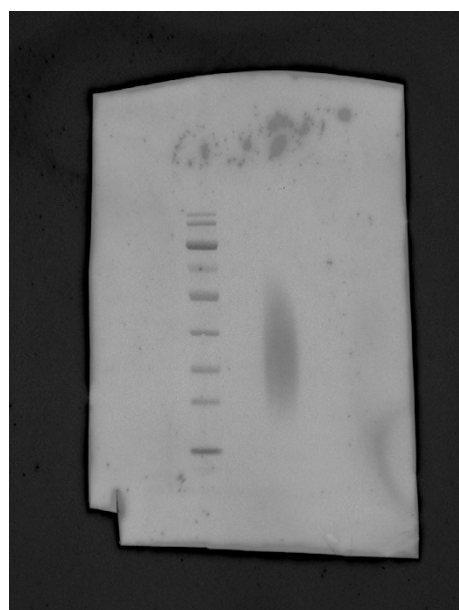

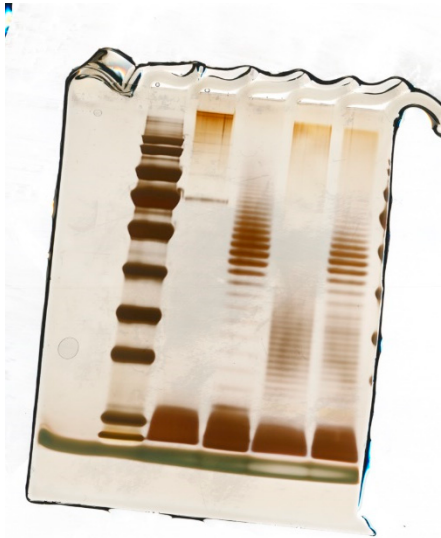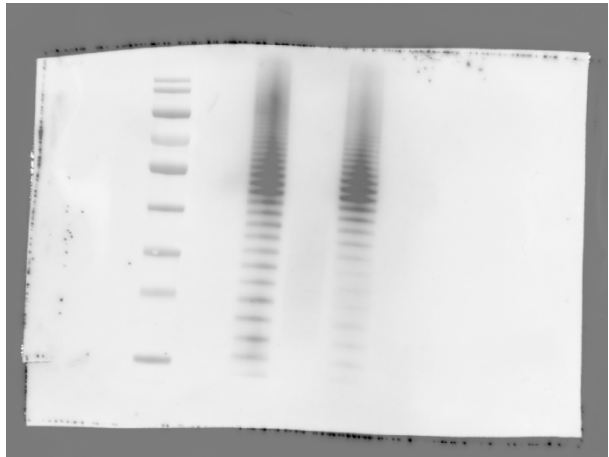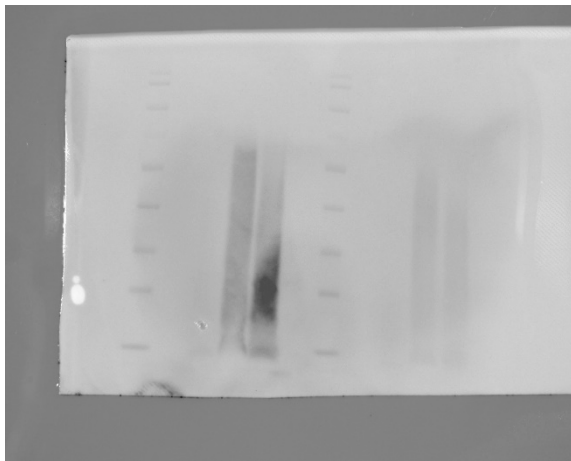

F2. B

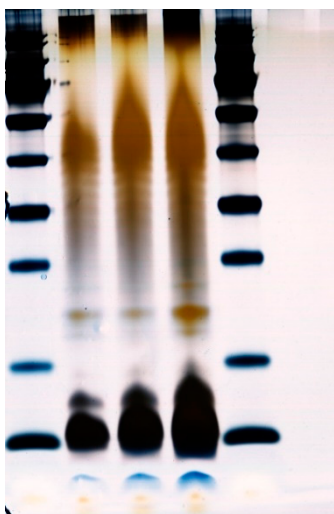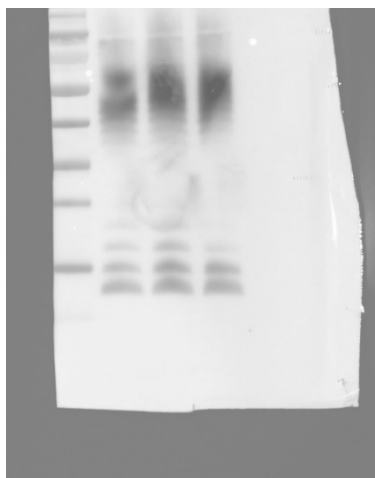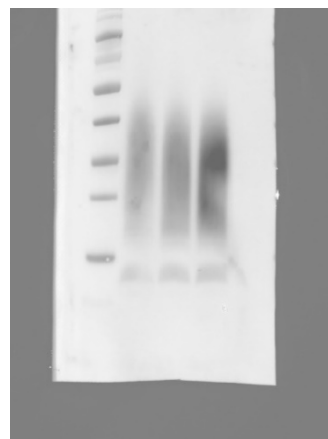

F3. A

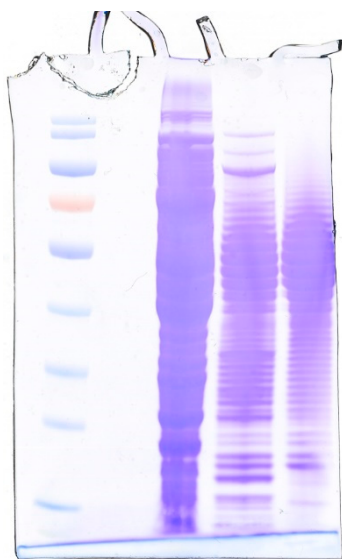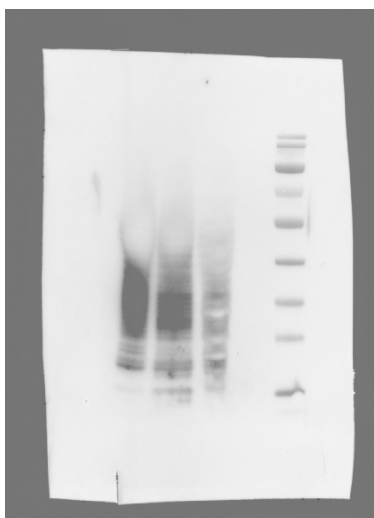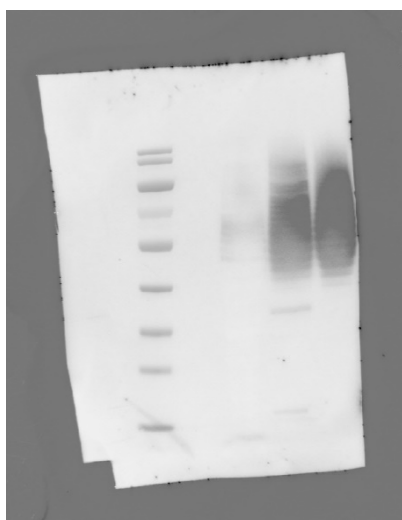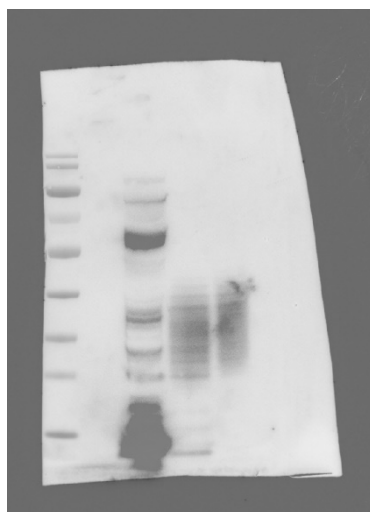

F4. B

Supplement: Supplementary file 1 [file life-15-00858-s001.zip › Figure S1. Original Figure.pdf]
